# Comparison of the effectiveness of lectures based on problems and traditional lectures in physiology teaching in Sudan

**DOI:** 10.1186/s12909-019-1799-0

**Published:** 2019-09-23

**Authors:** Nouralsalhin Abdalhamid Alaagib, Omer Abdelaziz Musa, Amal Mahmoud Saeed

**Affiliations:** 1 Department of Physiology, Faculty of Medicine, University of Khartoum, Khartoum, Sudan; 2 Department of Physiology, Faculty of Medicine, The National Ribat University, Khartoum, Sudan

**Keywords:** Traditional lectures, Lectures based on problems, Active learning, Medical education, Physiology

## Abstract

**Background:**

Lectures are one of the most common teaching methods in medical education. Didactic lectures were perceived by the students as the least effective method. Teaching methods that encourage self-directed learning can be effective in delivering core knowledge leading to increased learning. Problem based learning has been introduced as an active way of learning but it has some obstacles in developing countries where the intake is huge with minimum resources. This study introduces a new teaching approach: lectures based on problems (LBP) and evaluates their effectiveness compared to traditional lectures (TL) in physiology teaching.

**Methods:**

LBP and TL were applied in physiology teaching of medical students at University of Science and Technology during their study of introduction to physiology and respiratory physiology courses. Equal number of lectures was given as LBP and as TL in each course. Students were given quizzes at the end of each course which were used to compare the effectiveness of the two types of lectures. A questionnaire was used to assess students’ satisfaction about LBP and the perceived effects of the two methods on the students’ attitude and practice towards learning physiology.

**Results:**

In LBP the students have better attention (*P* = 0.002) and more active role (*P* = 0.003) than in TL. Higher percentage of students think that LBP stimulated them to use references more (*P* = 0.00006) and to use the lecture time more effectively (*P* = 0.0001) compared to TL. However, there was no significant difference between LBP and TL in the awareness of the learning objectives. About 64% of students think that LBP is more enjoyable and it improved their understanding of physiology concepts. Comparison of the students’ quiz marks showed that the means of the students’ marks in the introduction to physiology and respiratory courses were higher in the quizzes of LBP than in TL with a significant difference between them ((*P* = .000), (*P* = .006) respectively.

**Conclusions:**

LBP improved students’ understanding of physiology concepts and increased students’ satisfaction about physiology learning. LBP achieved some of the objectives of PBL with the minimum resources and it can be used to improve the effectiveness of the lectures.

## Background

Lectures are one of the most common teaching methods in medical education. It has been suggested that teaching methods that enhance engagement and encourage self-directed learning can be effective in delivering core knowledge and explaining difficult concepts leading to increased learning [1]. Transformation began with the introduction of problem-based learning (PBL) in some medical schools; more recently, lectures has increasingly been replaced by team-based learning.

Traditional, didactic lectures were perceived by the students as the least effective method used, yet involving students actively within the lecture time was regarded as a more effective learning tool [2]. Lectures have the advantage of sharing information with a large number of students and it can be effective in transmitting factual information [3]. Thus, lectures can be an effective teaching method when the lecture is given as large-group interactive learning sessions with discussion and frequent questions to students who have prepared in advance [4, 5]. Although the term “effective” has been widely used, the definitions of effectiveness is inescapably linked to the outcomes of educational activity through evaluation of the extent to which an activity approximates the achievement of its goals [6]. Generally some of the characteristics of effective teaching focus on teacher performance while others focus on student learning needs and outcomes [7]. Young and Shaw proposed six major dimensions of effective teaching: value of the subject, motivating students, a comfortable learning atmosphere, organization of the subject, effective communication, and concern for student learning [8]. Moreover, effective learning actively involves the student in metacognitive processes of planning, monitoring and reflecting. It is promoted by activity with reflection, collaboration for learning, learner responsibility for learning and learning about learning [9].

Lecturing, whether effective or not, is still the most commonly used learning method as it is an economical and practical method; especially when the number of students is large and the available resources are limited. For effective learning educationalists must change their use of the lecture time and make use of methods and techniques in which students are more active, communicating and collaborating for learning; with evaluation of the effectiveness of these methods by the students on their learning. Interactive lecture is an example of how knowledge about meaningful learning can be implemented in the lecture hall [10]. In interactive lecture students are asked to actively participate and process knowledge throughout the lecture. They also take an active part in contextualizing the content and directing the focus of the lecture towards areas they find difficult to understand [11]. Therefore, teachers can use the lecture to encourage students to construct their own understanding of concepts, relationships, and enhance application of theories by choosing suitable student centered learning approaches [12]. Chilwant compared structured interactive lectures with conventional lectures in two groups of second year medical students. The effect of two methods was evaluated by giving questionnaire and MCQs. Although their results showed no significant difference in average MCQ marks of two groups, students in the interactive group enjoyed being actively involved in the lecture which increased their engagement, attention during the lecture and stimulated their critical thinking [5]. Fyrenius *et. al.* presented a structure of organizing lectures into three phases, based on the theoretical prerequisites of meaningful learning like pre-understanding, relevance and active involvement [11]. The three phases are: introductory lecture (1–2 h), in-depth lecture (1–2 h) and application lecture (1–2 h) [11]. Moreover, lectures can be enriched by the use of educational media. Some studies showed that involvement of students in a large room or a lecture hall through voluntary participation in additional active learning exercises with aid of software [13] and use of game-like format of the review session and its custom-designed software, that combines interactivity, team learning and peer-to-peer instruction [14], resulted in an improvement in understanding and application of physiological concepts and enriched students’ learning experiences. Many Studies showed that students prefer learning approaches in which the students have more active role than the TL like case based learning [15], team based learning [16], small group discussion [17] and flipped classroom.

It is not only the method of teaching that affects the learning process; students’ own learning approaches influence their learning significantly**.** The learning approach adopted by students appears to be an important factor in determining both the quantity and the quality of their learning resulting in different learning outcomes [18]. Learning styles and learning approaches differ among medical students [19], and this could be partly attributable to their preferred learning style and partly to the context in which the learning takes place. Three basic approaches have been identified: surface, deep and strategic approaches [18]. The most desirable and successful is the deep approach in which students are motivated by an interest in the subject material. Their intention is to understand the material, to recognize its vocational relevance and to relate it to previous knowledge and personal experiences. The surface approach is rote learning in which students focus is on memorization pieces of information in isolation from the wider context motivated by either a desire to complete the course or a fear of failure. The main motivation of students using the strategic approach is achievement of high grades so they use either the surface or deep approach depending on what they feel would produce the most successful results [18]. They are much more influenced by the context than by the nature of the task itself. In TL students are passive recipient of information and have insufficient exposure to the content which encourages superficial learning. Abraham *et. al.* found that PBL promotes a deep approach to physiology learning and they suggested that physiology teaching outcomes could be improved through the use of the PBL teaching model [20]. Numerous studies that compared lecture-based learning (LBL) models to the PBL model showed certain advantages of PBL with respect to improving student abilities in active learning, critical thinking, communication skills, teamwork, critical thinking, peer-learning, self-learning and research skills [21–24]. However, a number of disadvantages of PBL were reported like: time constraints, inadequate resources, inconsistency in knowledge acquisition, inadequate contribution of clinicians and lack of required faculty training on PBL facilitation and required student preparation and motivation [24]. Moreover, PBL model seems difficult to apply in educational context with the large number of students and limited resources.

The main critique for LBL is the passive delivery of knowledge in a teacher centered approach; and students have insufficient exposure to the content which encourages superficial learning. However, interactive lectures proved to be effective in learning. For effective learning educators may need to use a variety of learning experiences [25]. A teaching model that combines the benefits of PBL and interactive lecture, by delivering lectures based on problems (LBP), may lead to better learning outcome with more active role of students during the lecture. This study introduces a new teaching approach LBP; to our knowledge they have not been used anywhere before. The aim of this study is to assess the effectiveness of LBP compared to TL and to evaluate the perceived effects of the two methods on the students’ attitude and practice towards learning physiology.

## Methods

### Study design

This is an interventional quasi study done in University of Science and Technology in Sudan in 2018. In this university, physiology is taught by the traditional curriculum and the duration of the lecture is 2 h. The study was done during the introduction to physiology course in the second semester of the first year and the respiratory course during the third semester in the second year for the same batch of medical students. In the introduction to physiology and the respiratory courses equal numbers of lectures were taught in a form of TL and LBP and both types were delivered by the same course instructor. Two of the authors contributed in teaching of the lectures by the two methods and each course was taught by a different instructor.

Steps of LBP:

- Step 1: Introducing the clinical problem to the whole class in 5 min.
- Step 2: Clarifying what is not clear in the scenario within the class in 5 min.
- Step 3: Paired student analysis of the problem for clues and key words and suggesting generally what the problem is about in 15 min. In this step students can use their books or mobile phones to search in the internet.
- Step 4: In the class, students share with the instructor what they have worked out in step 3. This takes about 10 min.
- Step 5: Students are given 10 min to formulate learning objectives based on the scenario and each pair of students should write down at least two learning objectives.
- Step 6: The instructor goes through the learning objectives in a 45–50 min lecture. The objectives of the problem can be taught in more than one lecture with reference to the same problem.
- Step 7: Later in the small groups tutorial after the students studied the learning resources they discuss the problem again and answer some short answer questions based on the scenario.
- Step 8: At the start of the next lecture, students are given quizzes on the previous one in 15–20 min. Each student has to solve these quizzes alone and then the answers are shared with the class.

### Inclusion criteria

All medical students in Batch 21 at University of Science and Technology. Each student should have attended at least 3 lectures of each of the TL and LBP in each course.

### Exclusion criteria

Students who did not attend 25% of the lectures in any of the two courses or did not attend the end of course test were excluded. Students who refused to participate or did not sign the informed consent were also excluded from the study.

### Sampling technique

By the end of the two courses, students were contacted by the investigator in the lecture hall. They were informed about the study and its objectives. To avoid measurement bias it was stated clearly that participation in the study will not affect their exam performance or grades by any means. Moreover, the data was collected using self– administered questionnaire that contains no names or identifiable information. An informed consent form attached with the questionnaire was passed by a teacher assistant to the students in the lecture hall after completion of the two courses. One hundred and forty six out of 183 students responded and agreed to fill the questionnaire.

To compare the effectiveness of the two methods quizzes were given to the students at the end of each course; in a form of multiple choice questions and short answer questions; covering the topics given as LBP and TL with equal weight. The quizzes tested some factual knowledge in addition to application of knowledge to explain some clinical signs and symptoms. Most questions asked were “how,” “what is the cause”, “what would happen if” or “explain” questions in addition to some questions to “define”, to “classify” or to draw a schematic diagram to explain a mechanism. All students were subjected to the same end of course quizzes with no difference between the groups regarding the kinds of knowledge tested. The results of these quizzes were used to compare the effectiveness of the two methods. The marks of the quizzes were taken from the secretary office in a form of excel sheet that doesn’t contain any names only the results of the whole class.

A questionnaire was filled by each participant to assess the perceived effect of the two methods on the students’ attitude and practice towards learning physiology through questions that determined the type of lectures in which the students had more active role and in which they were more aware to the learning objectives. Students were also asked about the type of lectures which stimulated them to use the lecture time more effectively, to use references and study resources and the type of lecture students think will enable them to score higher marks in the exam. Likert scale rating questions were used to assess students’ satisfaction about LBP and whether LBP improved their understanding of physiology concepts.

Results were saved and analyzed using SPSS version 23. Descriptive statistics were displayed in percentages and means ± SD. To evaluate the effectiveness of LBP, comparisons of students’ perception to certain items regarding LBP and TL were done using Z- test. According to Bonferroni criteria when assessing multiple tests for the same variable the level of significance should be adjusted by number of tests. Here the level of significance was adjusted as 0.05/6 = 0.0083 (where 6 is the number of tested items). Therefore, *P* value < 0.0083 is significant and *P* value < 0.0016 is considered highly significant. Comparison of the effectiveness of the two methods regarding students’ performance was done using independent t- test and a P value of ≤0.05 was taken as significant. Students’ satisfaction about LBP in physiology teaching was displayed as proportions.

## Results

One hundred and forty six out of 183 students responded and filled the questionnaire with a participation rate of 79.8%. Their age ranged between 17 and 24 years; 88.3% with age of 18–20 and the mean age was 18.7 ± 1.1 years. Two third of the class (62%) were females and 38% were males.

Comparison of the effect of the type of the lecture on students’ attitude and practice towards learning physiology is shown in Table 1.

**Table 1:** Comparisons of students’ responses to certain items regarding the two types of lectures

Item	LBP n%	TL n%	*P*-value
More aware to the learning objectives	48.9	29.5	0.0439
More active role	53.4	26.7	0.00321*
Better attention during the lecture	51.4	24.0	0.00282*
Use the lecture time more effectively and efficiently	58.2	24	0.00014**
Use references and study resources	58.2	21.9	0.00006**
Think will enable them to score higher exam marks	35.6	38.4	0.76799

* *P*-value < 0.0083 is significant. ** *P*- value < 0.00166 is highly significant

Results showed that in LBP students have significantly better attention (*P* = 0.002) and more active role (*P* = 0.003) than in TL. Fifty one percent of students think that they have better attention in LBP compared to 24% in the TL. Almost half the class (53.4%) have more active role in the LBP compared to 27.6% in the TL.

Higher percentage of students think that LBP stimulated them to use references more (*P* = 0.00006) and to use the lecture time more effectively (*P* = 0.0001) compared to TL with statistically highly significant difference between the two methods. Fifty eight percent of students think that they use the lecture time more effectively in the LBP and that the LBP stimulated them more to use the references and study resources to answer the questions more than the TL (Table 1). However, there was no significant difference between LBP and TL in the awareness of the learning objectives and the type of the lecture which students think will enable them to score higher marks in the exam (Table 1). Almost 20% of students found no difference between the two lectures methods regarding parameters reported in Table 1.

Reflections of students about LBP using the Likert scale rating (Table 2) showed that about 64% agreed that LBP improved their understanding of physiology concepts and that LBP are more enjoyable than TL. Almost two third of the class (64.4%) think that LBP should be continued and improved; 13% disagreed and 22.6% couldn’t decide.

**Table 2:** Assessment of students’ satisfaction about LBP

Variable	Strongly disagree	Disagree	Neutral	Agree	Strongly agree
LBP improved understanding of physiology concepts	4.1%	6.2%	25.3%	47.9%	16.4%
LBP are more enjoyable	1.4%	9.0%	24.8%	38.6%	26.2%
LBP are more satisfactory	4.1%	9.6%	27.4%	44.5%	14.4%
LBP should be continued and improved	4.8%	8.2%	22.6%	30.8%)	33.6%

Comparison of students’ quiz marks on the two methods was done using independent t-test (Table 3). The means of students’ marks in the introduction to physiology course (*n* = 101) and respiratory course (*n* = 146) were higher in the quizzes of LBP than in traditional lectures with a significant difference between the two methods ((*P* = .000), (*P* = .006) respectively).

**Table 3:** Comparison of students’ performance in the quizzes of the two types of lectures

Course	Lecture method	Number of students attended	Total Mark	Students' Marks (mean ± SD)	t-Test	*P*-value
Introduction to physiology	LBP	101	10	7.95 ± 1.65	9.35	.000**
	TL	101	10	5.72 ± 1.74		
Respiratory	LBP	146	2	9.68 ± 2.59	2.74	.006*
	TL	146	20	8.60 ± 4.02		

*P < 0.05 is significant, **P ≤ 0.005 is highly significant

In the introductory course 13/101 (12.87%) had the same score in the quizzes of the two methods. In the respiratory course 15/146 students (10.27%) score the same in the quizzes of the two methods.

## Discussion

The results of this study showed that about half the class thinks that they have more active role and better attention in the LBP compared to the TL. Although TL allow sharing a large body of content with a large number of students, they often promote passive and superficial learning. In TL the objectives of the lecture are shown at the start of the lecture and they are covered adequately by the teacher. However, students sitting passively may find difficulty in paying attention throughout the lecture. In LBP, when students are analyzing the problem for key words, searching the internet to know what is the problem about and during writing the learning objectives the instructor had to move throughout the lecture hall to monitor the class. It was rare to find a pair of students who were not seriously involved in the process. Throughout the lecture the instructor and the students try to link the lecture contents to the learning objectives and to the clinical scenario which contributed to the increase in students’ attention during the lecture. One of the most active interactions during LBP occurred when students shared the key words they have worked out and their suggestions of what the clinical problem could be about. This encouraged them to follow the lecture with enthusiasm and curiosity to find out whether they were right and to add what they missed. These results support the idea that the culture of the lecture is still acceptable by the students and that it can be an effective learning mechanism given that the students are engaged actively within the lecture [2].

In this study assessment of students attitude and practice towards learning physiology showed that a significantly higher percentage of students (58.2%) think that they use the lecture time more effectively in LBP than in TL and that LBP stimulated them more to use the references and study resources to learn physiology. By highly structuring the activities in LBP e.g. introducing a clinical problem, identifying difficult terminologies, analyzing the problem for clues and key words, formulating the learning objectives by the students and sharing the answers of the quizzes with the whole class, an active learning environment was created that enabled students to think, seek for information and use references, speak, and question freely. In LBP, the instructor may need to adopt an informal approach that promotes active learning through pair student interaction and discussion. Thus, the lecture became intellectually stimulating and challenging, as well as highly interactive. In LBP students are busy and participating actively in the lecture without significant loss of time. However, when more than hundred student talk at the same time the lecture hall may become noisy; that the instructor has to take control of the class and may need to use a signal to end conversations to be able to proceed [10]. TL seem easier for the instructor in controlling the class and managing the lecture time than LBP. In LBP highly structured classroom monitoring and time management is necessary to facilitate students’ interactions within the class and to proceed with the steps of LBP. Actually, the large number of students in the lecture hall is a major challenge for most educators who wish to innovate in teaching to make the lectures interactive and student centered. The use of student-centered active-learning instructional approaches, such as active- and inquiry-oriented learning in the classroom, improved student attitudes and increased learning outcomes relative to a standard lecture format [26, 27]. Students centered learning approach shifts the focus from teaching to learning and promotes a learning environment favorable of the metacognitive development necessary for students to become active independent learners and critical thinkers.

In this study 64.3% of students think that LBP improved their understanding of physiology concepts. In step7 of LBP, in the small groups tutorials after the students studied the learning resources and revised what they have learned in the lecture, they discuss the problem again and answer some questions based on the scenario. The interactions in this step with peers and facilitators give opportunities to the learners to apply what they have learned and allow exchange of information and construction of knowledge. In the lecture hall, students were given quizzes on the previous lesson and each student had to solve and then the answers were shared with the whole class. This can be considered a type of test-enhanced learning which facilitates retention of factual knowledge and it binds testing directly to teaching and the educational process. Larsen et al. reported that being tested on the material after reading it or hearing a lecture about a topic, enhance later retention of information and it is a better way to learn material than rereading it [28]. It was suggested that this technique may be particularly effective as students struggle to master complex and extensive sets of information, such as in physiology or pharmacology [28]. In addition, if students test themselves as a strategy for learning, they can discover their own areas of weakness and re-study material in a purposeful way. In LBP quizzes are given at the start of the next lecture about the previous lesson. It was suggested that for tests to enhance memory, they should be given relatively soon after learning and should be derived specifically from the information learned [29].

Sharing the answers of the quizzes with the whole class and providing feedback led, in most of the times, to discussion among the students in the class and this provided a good material for later discussion in the tutorial session. However, most likely it is the peer interaction rather than knowing the correct answer per se that promotes student learning [30] simply from peer influence of knowledgeable students on their neighbors. Furthermore, providing feedback enhances the benefits of testing by correcting errors and confirming correct responses [31]. This feedback and the short discussion that occurred in the lecture theatre allowed the instructor to have an idea about the depth of student understanding and the areas that need further clarification to be included in the tutorial’s questions.

We found that 58.9% of students think that LBP are more satisfactory. Two third of the students (about 64%) found LBP more enjoyable than TL and that LBP should be continued and improved. Moreover, comparison of the effectiveness of the two methods showed that the performance of students was significantly higher in the quizzes of LBP than those of TL in both the introduction to physiology and respiratory physiology courses. In LBP the use of quizzes and sharing and discussing the answers enhanced students’ learning and contributed to the better performance. However, the satisfaction most likely came from the problems introduced at the start of the lecture and the ability to apply knowledge of basic science on clinical setting which encourage deep learning approach. By using a clinical problem in LBP, students became clinically oriented and they appreciated the value of the basic information given at their level. Horne and Rosdahl showed that students found the case-based sessions better than TL format with respect to the overall learning experience, enjoyment of learning and increasing retention and ability to apply knowledge [32]. Basic science knowledge learned in the context of a clinical case is better comprehended and more easily applied by medical students than learning pure basic science knowledge [33, 34]. In medical education, physiology is not just the acquisition of fact and the understanding of the physiological mechanisms, but rather the ability to use this basic knowledge to understand the process of diseases, to explain some symptoms and signs, to suggest treatment and to acquire the skill of critical thinking and problem solving.

In spite of the fact that students’ performance was significantly better in the quizzes following LBP than the TL, assessment of students’ perception about their exam performance showed that higher percentage of students (38.4%) thinks that they will score better in the exam when they had TL compared to 35.6% who choose LBP and 26% think their score will be the same when they are taught by LBP or TL. This can be explained by the familiarity of the student to the didactic learning approach. Generally the means of learning in secondary education in Sudan put more responsibility on the teacher mainly ‘chalk and talk’ experience. Students in the first year in the university think that in the TL, they are given all the information needed to answer the exam questions unlike LBP in which they need to be self-learners and to solve questions and to use the references to reach the level of understanding needed to answer the exam questions. A second reason might be the anxiety felt by the students at time of exams. In this study students were more satisfied and enjoyed LBP more than TL; but when it comes to the exam students may become uncomfortable with LBP and may feel uncertain about what they have learned. They may think that they have wasted time on activities without knowing exactly what they have learned. Therefore, students may prefer more concrete and defined blocks of information given in the TL. They think their performance in the exam will be better when they know exactly the limits and boundaries of the subject they are going to be tested on.

In this study we found about 25% of students couldn’t decide and were neutral in all the investigated items regarding their satisfaction about LBP. This can be explained by the fact that students in this study were in the first year of their university study and they might be unaware of the benefits of the new active method and some students may be resistant to the active learning methods due to the increased self-learning and work outside the class. One of the studies that investigated student responses to active learning tasks showed that on initial exposure to the method, the majority of students found active methods strange, threatening and ineffectual. It is only with time and exposure to the method a change in students behavior may occur and students become comfortable and confident both with the method and their role [2]. However, most medical students later know that they must become lifelong learners to continue and succeed in their career. Therefore, students need the help of the educators to develop their skills in active self-directed learning and to practice more techniques of active learning.

### Limitation of the study

For LBP to be applied efficiently the teachers may need training on the steps of LBP. Like most of the active learning approaches time management is a challenge for educators who wish to use LBP. In this study students were given the clinical problem in the lecture. It would have been better if they were given the problem before the lecture to be prepared in advance. Also the use of clickers or colored cards would have improved sharing of answers for within the class questions.

## Conclusions

LBP is an effective active learning method which increased students’ satisfaction about physiology learning and improved students’ learning outcome in physiology. LBP achieved some of the objectives of PBL with the minimum resources and it can also be used by educators who want to improve the effectiveness of their lectures in medical schools that use the traditional curriculum.

The use of active student centered learning approaches in medical schools with large number of students should be evaluated by researches and should not be hindered by students’ resistance and complaint as they might not be familiar with these styles of learning and they may not be aware of the long term value of self- directed learning.

## Data Availability

The datasets used and/or analyzed during the current study are available from the corresponding author on reasonable request.

## Abbreviations

LBL: Lecture Based Learning
LBP: Lectures Based on Problems
PBL: Problem Based Learning
TL: Traditional Lectures

## References

[1] Wolff M, Wagner MJ, Poznanski S, Schiller J, Santen S (2015). Not another boring lecture: engaging learners with active learning techniques. J Emerg Med.

[2] Butler JA (1992). Use of teaching methods within the lecture format. Med Teach.

[3] McKeachie W (1994). Learning and cognition in the college classroom. Teaching tips: strategies, research and theory for college and university teachers.

[4] Schwartzstein RM, Roberts DH (2017). Saying goodbye to lectures in medical school—paradigm shift or passing fad?. N Engl J Med.

[5] Chilwant K (2012). Comparison of two teaching methods, structured interactive lectures and conventional lectures. Biomed Res.

[6] Belfield C, Thomas H, Bullock A, Eynon R, Wall D (2001). Measuring effectiveness for best evidence medical education: a discussion. Med Teach.

[7] Devlin M, Samarawickrema G (2010). The criteria of effective teaching in a changing higher education context. High Educ Res Dev.

[8] Young S, Shaw DG (1999). Profiles of effective college and university teachers. J High Educ.

[9] Watkins C, Lodge C, Whalley C, Wagner P, Carnell E (2002). Effective learning.

[10] Ebert-May D, Brewer C, Allred S (1997). Innovation in large lectures: teaching for active learning. Bioscience.

[11] Fyrenius A, Bergdahl B, Silén C (2005). Lectures in problem-based learning—why, when and how? An example of interactive lecturing that stimulates meaningful learning. Med Teach.

[12] Powell K (2003). Spare me the lecture. Nature.

[13] Carvalho H, West CA (2011). Voluntary participation in an active learning exercise leads to a better understanding of physiology. Adv Physiol Educ.

[14] Zakaryan V, Bliss R, Sarvazyan N (2005). Non-trivial pursuit of physiology. Adv Physiol Educ.

[15] Samuelson DB, Divaris K, De Kok IJ (2017). Benefits of case-based versus traditional lecture-based instruction in a preclinical removable prosthodontics course. J Dent Educ.

[16] Remington TL, Bleske BE, Bartholomew T, Dorsch MP, Guthrie SK, Klein KC (2017). Qualitative analysis of student perceptions comparing team-based learning and traditional lecture in a Pharmacotherapeutics course. Am J Pharm Educ.

[17] Joshi KP, Padugupati S, Robins M (2018). Assessment of educational outcomes of small group discussion versus traditional lecture format among undergraduate medical students. Int J Commun Med Public Health.

[18] Newble D, Entwistle N (1986). Learning styles and approaches: implications for medical education. Med Educ.

[19] Samarakoon L, Fernando T, Rodrigo C, Rajapakse S (2013). Learning styles and approaches to learning among medical undergraduates and postgraduates. BMC Med Educ.

[20] Abraham R, Vinod P, Kamath M, Asha K, Ramnarayan K (2008). Learning approaches of undergraduate medical students to physiology in a non-PBL-and partially PBL-oriented curriculum. Adv Physiol Educ.

[21] Kermaniyan F, Mehdizadeh M, Iravani S, MArkazi Moghadam N, Shayan S (2008). Comparing lecture and problem-based learning methods in teaching limb anatomy to first year medical students. Iranian J Med Educ.

[22] Enarson C, Cariaga-Lo L (2001). Influence of curriculum type on student performance in the United States medical licensing examination step 1 and step 2 exams: problem-based learning vs. lecture-based curriculum. Med Educ.

[23] Henderson S, Kinahan M, Rossiter E. Problem-based learning as an authentic assessment method. PG diploma in practitioner research projects. Dublin: DIT; 2018. https://arrow.dit.ie/ltcpgdprp/17/.

[24] Abdelkarim A (2018). Advantages and disadvantages of problem-based learning from the professional perspective of medical and dental faculty. EC Dent Sci.

[25] Silén C (2003). Responsibility and independence in learning–what is the role of the educators and the framework of the educational programme. Improving student learning: improving student learning–theory, research and practice (Oxford, the Oxford Centre for Staff and Learning Development).

[26] Preszler RW, Dawe A, Shuster CB, Shuster M (2007). Assessment of the effects of student response systems on student learning and attitudes over a broad range of biology courses. CBE Life Sci Educ.

[27] Armbruster P, Patel M, Johnson E, Weiss M (2009). Active learning and student-centered pedagogy improve student attitudes and performance in introductory biology. CBE Life Sci Educ.

[28] Larsen DP, Butler AC, Roediger HL (2008). Test-enhanced learning in medical education. Med Educ.

[29] Roediger HL, Karpicke JD (2006). The power of testing memory: basic research and implications for educational practice. Perspect Psychol Sci.

[30] Smith MK, Wood WB, Adams WK, Wieman C, Knight JK, Guild N (2009). Why peer discussion improves student performance on in-class concept questions. Science.

[31] Butler AC, Roediger HL (2008). Feedback enhances the positive effects and reduces the negative effects of multiple-choice testing. Mem Cogn.

[32] Horne A, Rosdahl J (2017). Teaching clinical ophthalmology: medical student feedback on team case-based versus lecture format. J Surg Educ.

[33] Patel VL, Evans D, Kaufman D (1990). Reasoning strategies and the use of biomedical knowledge by medical students. Med Educ.

[34] Patel VL, Groen G, Scott H (1988). Biomedical knowledge in explanations of clinical problems by medical students. Med Educ.

